# Supplementary material for: Pathotyping of Newcastle Disease Virus: a Novel Single BsaHI Digestion Method of Detection and Differentiation of Avirulent Strains (Lentogenic and Mesogenic Vaccine Strains) from Virulent Virus
Source: Microbiol Spectr. 2021 Dec 8;9(3):e00989-21. doi: 10.1128/spectrum.00989-21 (PMC8653816; doi:10.1128/spectrum.00989-21)
Supplement: SUPPLEMENTAL FILE 1 — Supplemental material. Download SPECTRUM00989-21_Supp_1_seq5.pdf, PDF file, 1.3 MB [file spectrum00989-21_supp_1_seq5.pdf]

Supplementary Table 1: Class I APMV-1 amino acid motif and their available sequence in NCBI with percentage (F gene cleavage site)

AA motif No.	AA motif of NDV (112-120)	No. of motif in NCBI	Percentage
1.	ERQERLIGA	29	8.12
2.	.Q.......	67	18.77
3.	.Q.G.....	4	1.12
4.	......V..	224	62.75
5.	KQ.......	1	0.28
6.	.Q..Q....	1	0.28
7.	...G..V..	6	1.68
8.	...D..V..	17	4.76
9.	V.....V..	2	0.56
10.	.K.G.....	1	0.28
11.	......L..	3	0.84
12.	G..G..V..	1	0.28
13.	GK.G.....	1	0.28
14.	Total	357	

Supplementary Table 2: Avirulent NDV Class II APMV-1, Nucleotide pattern, amino acid motif and their NUMBER, Nucleotide pattern available sequence in NCBI, countrywide, with percentage (F gene cleavage site)

Nucleotide pattern No.	Nucleotide pattern (334-360)	AA motif (112-120)	AA motif No	NPA NCBI*	Nucleotide pattern in a different country	Percentage
1.	GGG AGA CAG GGG CGC CTT TTA GGC GCC	GRQGRLLGA	1	2	Russia-2	0.23
2.	.A. ... .A ... ... A.. ... ..T	ERQGRLIGA	2	1	China -1	0.12
3.	... .A. ... ..A ... ..G A.. ... ..T	GKQGRLIGA	3	3	Argentina-3	0.35

4.	... .A. ... ..A ... ..G A.. ..T ..T	GKQGRLIGA	3	2	Argentina-2	0.23
5.	... ...C A..	GRQGRLIGA	4	1	Ireland-1	0.12
6.	... .AG ... ..A ... ... A.. ..A ...	GKQGRLIGA	3	1	Russia-1	0.12
7.	A.. ... ... AA. ... ... A..	RRQKRLIGA	5	7	India - 7	0.82
8.	... ... .C. ... ... A..	GRQARLIGA	6	1	India -1	0.12
9.	... .A. ... ..A ..T ... A..	GKQGRLIGA	3	83	Thailand-19, China -31, Australia-13, South Korea-10, USA-1, Nigeria-2, Tanzania-1, UK-3, Cameroon-1, Hungary-1, France-1	9.67
10.	.A. .AG ... ..A ..T ... A.. ..A ...	EKQGRLIGA	7	1	New Zealand-1	0.12
11.	... .A. ... ..A ..T ... A.. ..A ...	GKQGRLIGA	3	30	New Zealand-16, Australia-10, UK-4	3.50
12.	A.. ... ...T ... A..	RRQGRLIGA	8	11	New Zealand-1, Australia-9, UK-1	1.28
13.	A.A .A. ... ..A ... ... A.. ..A ...	RKQGRLIGA	9	14	Australia-12, UK-1, Iran-1	1.63
14.	... .A. ... ..A ... ... A.. ..A ...	GKQGRLIGA	3	19	Australia-19	2.21
15.	.A. .A. ... ..A ..T ... A..	EKQGRLIGA	7	2	Australia-2	0.23
16.	..A .A. ... ..A ... ... A.. ..A ...	GKQGRLIGA	3	2	Australia-2	0.23
17.	... ...A ..T ... A.. ..A ...	GRQGRLIGA	4	1	Australia-1	0.12
18.	... .A. ... ..A ... ... A.. ... ..T	GKQGRLIGA	3	118	China -48, South Korea-9, Japan-2, USA-11, Russia-6, Nigeria-5, UK-4, Slovakia-3, Finland-24, Sweden-3, Italy-3	13.75
19.	... .A. ... ... A.. ... ..T	GKQGRLIGA	3	36	China -33, South Korea-3	4.20
20.	... .A. ... ..A ... ... A.. ..T ..T	GKQGRLIGA	3	5	China -3, South Korea-1, Finland-1	0.58
21.	.A. .A. ... ... A.. ..T ..T	EKQGRLIGA	7	4	Hungary-1, Canada-2, USA-1	0.47
22.	... .A. ... ..A ... ... A..	GKQGRLIGA	3	19	Hungary-1, Ireland-1, USA-4, Russia-1, Brazil-2, Mexico-5, UK-3, China -1, Italy-1	2.21
23.	.A. .A. ... ..A ... ... A.. ... ..T	EKQGRLIGA	7	32	China -24, UK-1, Finland-3, Sweden-3, Italy-1	3.73
24.	... ...C ... ... A..	GRQGRLIGA	4	1	Italy-1	0.12
25.	... ... A.. ..T TT.	GRQGRLIGF	10	1	Italy-1	0.12

26.	... .A. ... ..A ... ..C A..	GKQGRLIGA	3	4	UK-3, Sweden-1	0.47
27.	.A. ... ... A..	ERQGRLIGA	2	1	UK-1	0.12
28.	... .A. ... .T ... A.. ..T ...	GKQGRLIGA	3	37	UK-1, USA-36	4.31
29.	... .A. ..A ..A ..T ... A..	GKQGRLIGA	3	2	UK-2	0.23
30.	... ... A.. ..T ...	GRQGRLIGA	4	13	Egypt-4, China -9	1.52
31.	A.. .A. ... ..A ..T ... A..	RKQGRLIGA	9	1	Tanzania-1	0.12
32.	.A. .AG ... ... A.. ..T ..T	EKQGRLIGA	7	74	USA-74	8.62
33.	.A. ... .A ..T ... A..	ERQGRLIGA	2	1	USA-1	0.12
34.	... .A. ... ...C A..	GKQGRLIGA	3	5	USA-4, Canada-1	0.58
35.	... .A. ... ..A ..T ... C..	GKQGRLLGA	13	4	Canada-4	0.47
36.	... .A. ... ... A.. ... ..G	GKQGRLIGA	3	3	China -3	0.35
37.	... ...A ... ... A.. ... ..T	GRQGRLIGA	4	1	China -1	0.12
38.	... .A. ..A ..A ... ... A..	GKQGRLIGA	3	3	China -1, Taiwan-1, Japan-1	0.35
39.	.A. .C. ... ... A..	ETQGRLIGA	11	1	China -1	0.12
40.	... .A. ... A.A ..T ... A..	GKQRRLIGA	12	1	China -1	0.12
41.	... ... A.. ..G ...	GRQGRLIGA	4	1	China -1	0.12
42.	... ... A..	GRQGRLIGA	4	309	China -155, USA-19, India-4, Pakistan-7, Malaysia-1, Taiwan-2, Turkey-2, Japan-2, Australia-3, South Korea-1, Iraq-2, Iran-2, Kazakhstan-8, Cameroon-7, Burkina Faso-5, Nigeria-12, Ethiopia-9, Tanzania-4, South Africa-6, Canada-2, UK-28, Argentina-1, Brazil-1, Mexico-9, Hungary-1, Ukraine-5, Germany-2, Belgium-5, France-1, Italy-3, Spain-1	36.01
43.	Total			858		

NP A NCBI* - Nucleotide pattern available in NCBI

Supplementary Table 3: Avirulent NDV Class II APMV-1, amino acid motif, Nucleotide pattern combination, and their available sequence in NCBI with percentage (F gene cleavage site)

AA motif No.	Avirulent NDV AA motif (112-120)	Nucleotide pattern combination	No. of Sequence in NCBI	Percentage
1.	GRQGRLLGA	1	2	0.23
**2.**	E.....I..	3	3	0.35
3.	.K....I..	17	372	43.36
4.	......I..	7	327	38.11
5.	R..K..I..	1	7	0.82
6.	...A..I..	1	1	0.12
7.	EK....I..	5	113	13.17
8.	R.....I..	1	11	1.28
9.	RK....I..	2	15	1.75
10.	......I.F	1	1	0.12
11.	ET....I..	1	1	0.12
12.	.K.R..I..	1	1	0.12
13.	.K.......	1	4	0.47
14.	total		858	

Supplementary Table 4: Virulent NDV Class II APMV-1, Nucleotide pattern, amino acid motif and their NUMBER, Nucleotide pattern available sequence in NCBI, countrywide, with percentage (F gene cleavage site)

**Nucleotide pattern No.**	**Nucleotide pattern (334-360)**	**AA motif (112-120)**	**AA motif No**	**NPA NCBI ***	**Nucleotide pattern in a different country**	**Percentage**
1.	AGG AGA CAG AAA CGC TTT ATA GGT GCC	RRQKRFIGA	1	309	China-23, USA -6, Russia-4, UK-28, India -3, Pakistan-47, Malaysia-10, Indonesia-7, Taiwan-6, Japan-21, Australia-1, South Korea-5, Iran-1, Iraq-6, Kuwait-1, United Arab Emirates-1, Israel-10, Lebanon-1, Mexico-17, Argentina-1, Peru-1, Central African Republic-1, Bulgaria-4, Croatia-1, Hungary-41, Sweden-1, Germany-1, Czech Republic-1, Belgium-1, Italy-4, Mali-1, Burkina Faso-5, Benin-11, Niger-4, Nigeria-32, South Africa-1	11.96
2.	... ...T	RRQKRFIGA	1	9	Pakistan-6, Sweden -1, UK-1, Egypt-1	0.35
3.	... ... .G.	RRQRRFIGA	2	75	Pakistan-6, India -1, Singapore-1, Japan-2, Australia-1, China-16, Russia-7, Nigeria-1, UK-10, Bulgaria-3, Hungary-18, Finland-2, Belgium-1, Italy-6	2.90
4.	..A .A. ... .T	RKQKRFIGA	3	4	Pakistan-4	0.15
5.	... ... A.A	RRKKRFIGA	4	10	Vietnam-1, Taiwan-5, China-2, USA -2	0.39
6.	... ... .G. ..T	RRQRRFIGA	2	78	Malaysia-8, Kazakhstan-15, Kyrgyzstan-6, Iran-25, Turkey-6, Russia-1, UK-6, Bulgaria-3, Hungary-6, Finland-1, Sweden-1	3.02
7.	... ..G ... .GG	RRQRRFIGA	2	2	Malaysia-1, Italy-1	0.08
8.	... .A ... ...T	RRQKRFIGA	1	618	China-509, Malaysia-4, Singapore-3, South Korea-69, Iran-1, Saudi Arabia-1, Israel-5, Sudan-5, South Africa-4, UK-3, Macedonia-9, Serbia-2, Hungary-1, Ukraine-1,	23.92

					Italy-1	
9.	... .A	RRQKRFIGA	1	120	Malaysia-1, Taiwan-27, Japan-23, South Korea-1, Kazakhstan-6, Kyrgyzstan-2, Egypt-1, Togo-4, Niger-1, Nigeria-2, Ivory Coast-2, Ukraine-10, China-40	4.64
10.	C.T C.T .GT ... ..T ... ..T ..C ..G	RRRKRFIGA	5	7	Malaysia-7	0.27
11.	... ... .G.	RRRKRFIGA	5	39	Malaysia-15, Indonesia-3, South Korea-6, Iran-3, Nigeria-1, Bulgaria-2, China-9	1.51
12.	... ...T	RRQKRFIGA	1	18	Malaysia-1, USA -1, China-3, Taiwan-1, Japan-1, Australia-2, Iran-1, Benin-1, Nigeria-5, UK-2	0.70
13.	.A. ..G ... .A	KRQKRFIGA	6	2	China-1, Singapore-1	0.08
14.	... .A ... ...C	RRQKRFIGA	1	1	Singapore-1	0.04
15.	.A.	KRQKRFIGA	6	12	Greece-1, China-6, Singapore-1, Hungary-4	0.46
16.	... ...G ... ...T	RRQKRFIGA	1	2	Indonesia-2	0.08
17.	... .A .G.	RRQRRFIGA	2	4	Taiwan-1, India-1, Russia-2	0.15
18.	.AA C.G ... ..G ..T ... G.. ..G ..A	KRQKRFVGA	15	4	China-1, Japan-2, Ireland-1	0.15
19.	... ...G	RRQKRFIGA	1	20	Japan-3, India-3, Australia-3, China-7, Slovenia-1, Hungary-1, Belgium-1, Italy-1	0.77
20.	G.. ..G	GRQKRFIGA	7	68	China-1, India-9, UK-33, Ireland-5, Hungary-18, Belgium-1, Italy-1	2.63
21.	... ..G	RRQKRFIGA	1	41	China-1, Japan-1, Iraq-2, USA-9, Russia-2, Canada-2, UK-6, Sweden-1, Tanzania-1, Hungary-15, Germany-1	1.59
22.	... ..G A.. ..G	RRKKRFIGA	4	69	USA-21, Canada-1, Panama-1, Austria-2, Hungary-13, Slovakia-2, Finland-3, Denmark-1, Germany-3, France-1, Ireland-2, South Africa-10, Japan-6, UK-3	2.67
23.	... ... .G. ... ... G..	RRQRRFVGA	8	5	Austria-1, USA-1, UK-3	0.19
24.	... ... .GA	RRRKRFIGA	5	9	South Korea-5, Togo-2, Benin-1, China-1	0.35
25.	... ... A.A ... ...T	RRKKRFIGA	4	3	South Korea-2, China-1	0.12
26.	... .A .G. ... ...T	RRQRRFIGA	2	4	South Korea-1, China-3	0.15

27.	... .A ... ... G.. ... ..T	RRQKRFVGA	9	2	South Korea-2,	0.08
28.	... ... .GA ... ...T	RRRKRFIGA	5	14	South Korea-1, China-13	0.54
29.	.A. ... .G	KRQKRFIGA	6	42	Kazakhstan-3, Austria-1, Russia-36, Nigeria-2	1.63
30.	... .A ... ..T ... ...T	RRQKRFIGA	1	89	Iraq-2, Iran-19, Israel-34, China-26, USA-3, Egypt-5	3.44
31.	... .A ..G ..T ... ...T	RRQKRFIGA	1	1	Iran-1,	0.04
32.	... ..G ... ..G	RRQKRFIGA	1	127	Turkey-2,UAE-3, Hungary-9, South Africa-6, China-28, Russia-3, UK-26, Belgium-11, France-2, Italy-6, Portugal-1, Ireland-6, Macedonia-1, Slovenia-9, Slovakia-10, Finland-2, Denmark-2	4.91
33.	... ..G ... ..G ..T	RRQKRFIGA	1	14	UAE-5, Sweden-1, Uruguay-1, Saudi Arabia-1, UK-2, Argentina-1, Hungary-2, Russia-1	0.54
34.	C.. ..G ... ..G ..T	RRQKRFIGA	1	1	UAE-1	0.04
35.	... ...G ... ... G..	RRQKRFVGA	9	4	Israel-2, UK-1, Hungary-1	0.15
36.	... .A ... ... T.. ... ..T	RRQKRFLGA	10	2	Israel-1, China-1	0.08
37.	... ..G A.A	RRKKRFIGA	4	1	Turkey-1	0.04
38.	... ..G ... ..G ... ..C	RRQKRFIGA	1	17	China-5, Belgium-3, Macedonia-9	0.66
39.	... ... .G. ... ...T	RRQRRFIGA	2	16	Sudan-1, Bulgaria-2, India-12, Nigeria-1	0.62
40.	... ... G..	RRQKRFVGA	9	112	South Korea-2, Brazil-1, Belize-2, Mexico-2, Argentina-1, Bulgaria-10, Croatia-27, Slovenia-9, Italy-10, Hungary-28, USA-8, UK-7, Tanzania-3, Nigeria-2,	4.33
41.	... .A ... ... G..	RRQKRFVGA	9	6	Croatia-4, Hungary-1, UK-1	0.23
42.	G.A ..G	GRQKRFIGA	7	19	UK-1, Slovenia-1, Italy-6, Hungary-11	0.74
43.	... ...C G..	RRQKRFVGA	9	4	Ethiopia-3, Hungary-1	0.15
44.	... ..G ... ..G ... ...A ...	RRQKRFIGA	1	5	UK-1, Hungary-3, Germany-1	0.19
45.	... ..G ... .G	RRQKRFIGA	1	4	UK-1, Hungary-1, Finland-2	0.15

46.	... ... T..	RRQKRFLGA	10	3	Hungary-2, Peru-1	0.12
47.	... ...A	RRQKRFIGA	1	2	Hungary-1, South Africa-1	0.08
48.	... ..G ..A	RRQKRFIGA	1	22	Hungary-1, China-21	0.85
49.	.A. ..G	KRQKRFIGA	6	1	Hungary-1	0.04
50.	... ...T ..C ..T	RRQKRFIGA	1	1	Hungary-1	0.04
51.	... ...T ... G..	RRQKRFVGA	9	4	Hungary-1, UK-1, USA-2	0.15
52.	..A ... .G.	RRRKRFIGA	5	4	South Africa-1, Hungary-3	0.15
53.	..A ..G ... ..G	RRQKRFIGA	1	1	Austria-1	0.04
54.	..A C.. ... ...A	RRQKRFIGA	1	20	Belgium-1, Nigeria-14, Niger-5	0.77
55.	... ..G .G. ..G ..T	RRRKRFIGA	5	6	Belgium-1, Nigeria-5	0.23
56.	..A C.. .G. ... ..T ... G.. ... ..G	RRRKRFVGA	12	4	Belgium-1, Nigeria-3	0.15
57.	... ...C	RRQKRFIGA	1	19	China-4, South Africa-9, Nigeria-3, Belgium-1, Israel-1, UK-1	0.74
58.	... ..G A..	RRKKRFIGA	4	4	Italy-3, UK-1	0.15
59.	... ..G .G. ..G	RRRKRFIGA	5	2	Italy-2	0.08
60.	G.A ..G ..A	GRQKRFIGA	7	1	Italy-1	0.04
61.	... .A ..G	RRQKRFIGA	1	7	Italy-1, Ivory Coast-1, Mali-3, Mauritania-1, Vietnam-1	0.27
62.	... .A ..G ..T	RRQKRFIGA	1	1	Italy-1	0.04
63.	.A. ..G ... ..G ... ... A..	KRQKRFIGT	19	1	Italy-1	0.04
64.	... ... .G. ..T ... ...T	RRQRRFIGA	2	3	Russia-1, Finland-1, Spain-1	0.12
65.	... ..G ... ..G ... ...T	RRQKRFIGA	1	3	Russia-1, Portugal-2	0.12
66.	.A. ..G ... ..G	KRQKRFIGA	6	5	Russia-2, Portugal-1, Finland-1, China-1	0.19
67.	G.. ..G ..A	GRQKRFIGA	7	14	UK-14	0.54
68.	G.. ..G ... .T	GRQKRFIGA	7	2	UK-2	0.08
69.	... ...T ... G.. ... ..T	RRQKRFVGA	9	54	UK-9, USA-10, Canada-35	2.09

70.	... ...C ... .T	RRQKRFIGA	1	2	UK-1, Turkey-1	0.08
71.	... ..G A.A ..G	RRKKRFIGA	4	1	UK-1	0.04
72.	... ..G ... .G. ... ... G..	RRQRRFVGA	8	1	UK-1	0.04
73.	G.. ..G ... ...C	GRQKRFIGA	7	1	UK-1	0.04
74.	.A. ... ... .G. ..T	KRQRRFIGA	11	1	UK-1	0.04
75.	... ... .G. ..T ... G..	RRQRRFVGA	8	1	UK-1	0.04
76.	..A ... .GA ... ... G.. ... ..A	RRRKRFVGA	12	4	Mali-1, Burkina Faso-3	0.15
77.	... ..G ... ...C	RRQKRFIGA	1	12	Niger-1, Nigeria-7, Benin-4	0.46
78.	... ..G A.. ..G ..T	RRKKRFIGA	4	3	Nigeria-3	0.12
79.	... ..G A.. ..G ..T ... ...T	RRKKRFIGA	4	2	Nigeria-2	0.08
80.	... ...A ...	RRQKRFIGA	1	1	Nigeria-1	0.04
81.	..A C.. .G. ... ..T ... G.. ... ..A	RRRKRFVGA	12	30	Nigeria-29, Benin-1	1.16
82.	... ..G .G. ... .C	RRRKRFIGA	5	9	Nigeria-9	0.35
83.	..A C.. ... .T ... G.. ... ..A	RRQKRFVGA	9	11	Nigeria-11	0.43
84.	..A C.. ... ...G ..A	RRQKRFIGA	1	1	Nigeria-1	0.04
85.	..A ... ...T	RRQKRFIGA	1	1	Nigeria-1	0.04
86.	... ... A.. ..G ..T	RRKKRFIGA	4	2	Nigeria-1, usa-1	0.08
87.	C..	RRQKRFIGA	1	2	Burundi-2	0.08
88.	... ..G ... .G. ..T	RRQRRFIGA	2	1	Tanzania-1	0.04
89.	... .A ... ...A	RRQKRFIGA	1	16	China-3, South Africa-13,	0.62
90.	... .A ... ...A ..A	RRQKRFIGA	1	1	South Africa-1	0.04
91.	..A	RRQKRFIGA	1	5	South Africa-4, Hungary-1	0.19
92.	... ... .GA ... ...A	RRRKRFIGA	5	1	South Africa-1	0.04
93.	... ..G .G. .G. ... ... G..	RRRRRFVGA	13	3	Madagascar-3	0.12
94.	... .A ... ..T ... G.. ... ..T	RRQKRFVGA	9	1	Canada -1	0.04
95.	.A. ... ..A ..G	KRQKRFIGA	6	5	Russia-5	0.19

96.	.AA C.. ... ..G	KRQKRFIGA	6	2	Russia-2	0.08
97.	.A. C.. ... ..G	KRQKRFIGA	6	24	Russia-24	0.93
98.	.A. ... A.. ..G	KRKKRFIGA	14	2	Russia-1, usa-1	0.08
99.	.A. C..	KRQKRFIGA	6	1	Russia-1	0.04
100.	.A. ... .G ... ...A ...	KRQKRFIGA	6	1	Russia-1	0.04
101.	... ... A.. ..G	RRKKRFIGA	4	43	USA-43	1.66
102.	.A. ... ...T ... G.. ... ..T	KRQKRFVGA	15	13	USA-13	0.50
103.	.A. ... ...T ... G.G ... ..T	KRQKRFVGA	15	2	USA-2	0.08
104.	... ... A..	RRKKRFIGA	4	1	USA-1	0.04
105.	... ..G ..A ... ...T	RRQKRFIGA	1	5	USA-1, Peru-1, China-3	0.19
106.	... .A ... .C ... .T	RRQKRFIGA	1	66	China-66	2.55
107.	.A. ..G ... .G	KRQKRFIGA	6	25	China-25	0.97
108.	... ..G ... .G.	RRQRRFIGA	2	42	China-42	1.63
109.	... .A ... ..A ... ...T	RRQKRFIGA	1	3	China-3	0.12
110.	... ... A.A ... ..A ... ...T	RRKKRFIGA	4	2	China-2	0.08
111.	... .A ... ..T ..C ... .T	RRQKRFIGA	1	1	China-1	0.04
112.	... .A ..G ... ...T	RRQKRFIGA	1	2	China-2	0.08
113.	... .A ... ..T ..C	RRQKRFIGA	1	2	China-2	0.08
114.	.A. ..G ... .G ..C	KRQKRFIGA	6	3	China-3	0.12
115.	.A. ..G ..A ... ..G	KRQKRFIGA	6	1	China-1	0.04
116.	G.. ... ... GGG ... ...T	GRQGRFIGA	16	1	China-1	0.04
117.	G.. ... ..A ... ...T	GRQKRFIGA	7	1	China-1	0.04
118.	... .A ... ...C ..T	RRQKRFIGA	1	3	China-3	0.12
119.	... .A ... .C ... ..C ..T	RRQKRFIGA	1	1	China-1	0.04
120.	... ..G .GA ..G ... ..C	RRRKRFIGA	5	6	China-6	0.23
121.	... ..G ..A ... ..T ... ...T	RRQKRFIGA	1	1	China-1	0.04

122.	... ... A.A ... ..T ... ...T	RRKKRFIGA	4	1	China-1	0.04
123.	G..	GRQKRFIGA	7	2	Argentina-2	0.08
124.	... ...A	RRQKRFIGA	1	1	Mexico-1	0.04
125.	C.T C.T ... .T ... ..T ..C ..G	RRQKRFIGA	1	1	Malaysia-1	0.04
126.	... ...C G.. ... ..T	RRQKRFVGA	9	2	South Korea-2	0.08
127.	... .A ... ..T ... ... A.. ..T	RRQKRFISA	17	1	Iran-1	0.04
128.	... ... .G. ... .C	RRRKRFIGA	5	1	Hungary-1	0.04
129.	... ...G ... ... G.. A.. ...	RRQKRFVSA	18	1	Hungary-1	0.04
130.	..A ... .G. ... ..T ... G.. ... ..A	RRRKRFVGA	12	1	Nigeria-1	0.04
131.	..A C.G ... .T ... G.. ... ..A	RRQKRFVGA	9	1	Nigeria-1	0.04
132.	..A C.. .G. ..G ..T ... G.. ... ..A	RRRKRFVGA	12	1	Nigeria-1	0.04
133.	..A ... .G. ... .C G..	RRRKRFVGA	12	1	Ethiopia-1	0.04
134.	... ... G.. ... ..T	RRQKRFVGA	9	1	China-1	0.04
135.	total			2584		

NP A NCBI* - Nucleotide pattern available in NCBI

Supplementary Table 5: Virulent NDV Class II APMV-1, amino acid motif, Nucleotide pattern combination, and their available sequence in NCBI with percentage (F gene cleavage site)

AA motif No.	Virulent AA motif (112-120)	Nucleotide pattern combination	No. of Sequence in NCBI	Percentage
1.	RRQKRFIGA	46	1640	61.56
2.	...R.....	9	253	9.50
3.	.K.......	1	4	0.15
4.	..K......	13	142	5.33
5.	..R......	11	99	3.72
6.	K........	13	125	4.69

7.	G........	8	113	4.24
8.	...R..V..	3	9	0.34
9.	......V..	12	204	7.66
10.	......L..	2	5	0.19
11.	K..R.....	1	1	0.04
12.	..R...V..	6	41	1.54
13.	..RR..V..	1	3	0.11
14.	K.K......	1	2	0.08
15.	K.....V..	3	19	0.71
16.	G..G.....	1	1	0.04
17.	.......S.	1	1	0.04
18.	......VS.	1	1	0.04
19.	K.......T	1	1	0.04
20.	Total		2664 Virulent 2584 + 80 mesogenic	

Supplementary Table 6: Mesogenic NDV Class II APMV-1, Nucleotide pattern, amino acid motif and their NUMBER, Nucleotide pattern available sequence in NCBI with percentage (F gene cleavage site)

**Nucleotide pattern No.**	**Nucleotide pattern (334-360)**	**AA motif (112-120)**	**AA motif No**	**NP A NCBI ***	**Percentage**
1.	AGG AGA CAG AAA CGC TTT ATA GGC GCC	RRQKRFIGA	1	29	35.36585
2.	... ... .GG ..T	RRQRRFIGA	2	22	26.82927
3.	... ... .GG ..A	RRQRRFIGA	2	1	1.219512

4.	... ... GGG ..T	RRQGRFIGA	3	1	1.219512
5.	... ...G ... ... G..	RRQKRFVGA	4	1	1.219512
6.	... ... G..	RRQKRFVGA	4	1	1.219512
7.	... .A	RRQKRFIGA	1	2	2.439024
8.	... ... .G.	RRQRRFIGA	2	6	7.317073
9.	... ...A	RRQKRFIGA	1	7	8.536585
10.	... ... .CA	RRQKPFIGA	5	1	1.219512
11.	G.A ..G ... ...C	GRQKRFIGA	6	4	4.878049
12.	... ... .G. ..T ... G..	RRQRRFVGA	7	1	1.219512
13.	G.A ..G	GRQKRFIGA	6	1	1.219512
14.	... ..G	RRQKRFIGA	1	2	2.439024
15.	... ... .G. ... ... G..	RRQRRFVGA	7	1	1.219512
16.	.A. ... .G	KRQKRFIGA	8	1	1.219512
17.	... ..G ..A ..G	RRQKRFIGA	1	1	1.219512

P A NCBI* - Nucleotide pattern available in NCBI

Supplementary table 7: Mesogenic NDV Class II APMV-1, amino acid motif

**AA motif No.**	**Mesogenic NDV AA motif (112-120)**
1.	RRQKRFIGA
2.	...R.....
3.	...G.....
4.	......V..
5.	....P....
6.	G........
7.	...R..V..
8.	K........

Supplementary Table 8: Comparison of Nucleotide pattern, Amino acid motif, ICPI and *BSAH*1 digestion

GenBank no.	Name Of Isolate	Nucleotide pattern (334-360)	Amino acid (112-120)	ICPI	*BSA H*1 digestion	Reference
APMV1/CT/ Sask/1479/90	AF448223	AGGAGACAGAAACGTTTTGTAGGTGCT	RRQKRFVGA	1.55	ND	Weingartl et al 2003 (1)
APMV1/CT/ Sask/2585/92	AF448844	AGGAGACAGAAACGTTTTGTAGGTGCT	RRQKRFVGA	1.6	ND	Weingartl et al 2003 (1)
APMV1/CT/ Man/3298/92	AF448843	AGGAGACAAAAACGTTTTGTAGGTGCT	RRQKRFVGA	1.3	ND	Weingartl et al 2003 (1)
APMV1/CT/ Ont/2150/95	AF448486	AGGAGACAGAAACGTTTTGTAGGTGCT	RRQKRFVGA	1.6	ND	Weingartl et al 2003 (1)
APMV1/CT/ Ont/2575/95	AY063493	AGGAGACAGAAACGTTTTGTAGGTGCT	RRQKRFVGA	1.88	ND	Weingartl et al 2003 (1)
APMV1/CT/ Sask/2035/95	AF448845	AGGAGACAGAAACGTTTTGTAGGTGCT	RRQKRFVGA	1.61	ND	Weingartl et al 2003 (1)
APMV1/CT/ Ont/39/96	AF448842	AGGAGACAGAAACGTTTTGTAGGTGCT	RRQKRFVGA	1.15	ND	Weingartl et al 2003 (1)
APMV1/CT/ Ont/48/96	AY063492	AGGAGACAGAAACGTTTTGTAGGTGCT	RRQKRFVGA	1.66	ND	Weingartl et al 2003 (1)
APMV1/CT/ Sask/3-1125/98	AY063494	AGGAGACAGAAACGTTTTGTAGGTGCT	RRQKRFVGA	1.56	ND	Weingartl et al 2003 (1)
APMV1/CT/ Alb/35/99	AF448841	AGGAGACAGAAACGTTTTGTAGGTGCT	RRQKRFVGA	1.07	ND	Weingartl et al 2003 (1)
APMV1/CT/ Ont/378/00	AY063123	AGGAGACAGAAACGTTTTGTAGGTGCT	RRQKRFVGA	1.3	ND	Weingartl et al 2003 (1)
2K36/Peacock/Chennai/India/2009	FJ871120	AGGAGACAGAGACGCTTTATAGGCGCT	RRQRRFIGA	1.71	ND	Vijayarani et al 2010 (2)
2736/00	AF520965	AGGAGGAAGAAACGCTTTATAGGTGCC	RRKKRFIGA	1.30	ND	Terregino et al., 2003 (3)
7007/00	AF520972	AGGAGGCAGAAGCGCTTTATAGGTGCC	RRQKRFIGA	0.72	ND	Terregino et al., 2003 (3)
SGM/01	DQ227248	AGGAGACAAAAACGCTTTATAGGTGCT	RRQKRFIGA	1.78	ND	Qin et al., 2008 (4)
DQ363531	JS04/04	AGGAGACAAAAACGCTTTATAGGTGCT	RRQKRFIGA	1.95	ND	Qin et al., 2008 (4)
DQ363535	SF/02	AGGAGACAAAAACGCTTTATAGGTGCT	RRQKRFIGA	1.81	ND	Qin et al., 2008 (4)

DQ363536	TJ05/05	AGGAGACAAAAACGCTTTATAGGTGCT	RRQKRFIGA	1.47	ND	Qin et al., 2008 (4)
DQ227251	SKY/03	AGGAGACAAAAACGCTTTATAGGTGCT	RRQKRFIGA	1.89	ND	Qin et al., 2008 (4)
DQ417110	JS01/01	AGGAGACAAAAACGCTTTATAGGTGCT	RRQKRFIGA	1.91	ND	Qin et al., 2008 (4)
DQ363534	JS03/03	AGGAGACAAAAACGCTTTATAGGTGCT	RRQKRFIGA	1.60	ND	Qin et al., 2008 (4)
DQ227253	SPY/03	AGGAGACAAAAACGCTTTATAGGTGCT	RRQKRFIGA	1.92	ND	Qin et al., 2008 (4)
DQ228922	SL/03	AGGAGACAAAAACGCTTTATAGGCGCT	RRQKRFIGA	1.94	ND	Qin et al., 2008 (4)
DQ227247	SDD/01	AGGAGACAAAAACGCTTTATAGGCGCT	RRQKRFIGA	2.00	ND	Qin et al., 2008 (4)
DQ227249	SQD/04	AGGAGACAAAAACGCTTTATAGGCGCT	RRQKRFIGA	1.96	ND	Qin et al., 2008 (4)
DQ363533	SCL/03	AGGAGACAAAAACGCTTTATAGGTGCT	RRQKRFIGA	1.91	ND	Qin et al., 2008 (4)
DQ363530	WHZ/03	AGGAGACAAAAACGCTTTATAGGTGCT	RRQKRFIGA	1.94	ND	Qin et al., 2008 (4)
DQ368683	GD/05	AGGAGACAAAAACGCTTTATAGGTGCT	RRQKRFIGA	1.23	ND	Qin et al., 2008 (4)
DQ417111	SSX/03	AGGAGACAAAAACGCTTTATAGGTGCT	RRQKRFIGA	1.95	ND	Qin et al., 2008 (4)
DQ363538	Lye/01	AGGAGGCAAAAACGCTTTATAGGTGCC	RRQKRFIGA	1.88	ND	Qin et al., 2008 (4)
DQ363537	Jlan/04	AGGAGACAAAAACGCTTTATAGGTGCC	RRQKRFIGA	1.88	ND	Qin et al., 2008 (4)
DQ227254	SWS/03	AGGAGACAGAAACGCTTTATAGGTGCC	RRQKRFIGA	1.91	ND	Qin et al., 2008 (4)
DQ227246	JS02/99	AGGAGACAGAAACGCTTTATAGGTGCC	RRQKRFIGA	1.91	ND	Qin et al., 2008 (4)
DQ858357	YG/03	AGGAGACAGAAACGCTTTATAGGTGCC	RRQKRFIGA	1.85	ND	Qin et al., 2008 (4)
DQ417113	PB01/96	AAGAGGCAGAAACGGTTTATAGGTGCC	KRQKRFIGA	1.65	ND	Qin et al., 2008 (4)
DQ227252	SBD/02	AGGAGGCAGAGACGCTTTATAGGTGCC	RRQRRFIGA	1.70	ND	Qin et al., 2008 (4)
DQ227244	TJ03/03	AGGAGGCAGAGACGCTTTATAGGTGCC	RRQRRFIGA	1.60	ND	Qin et al., 2008 (4)

DQ858356	JS06/03	AGGAGGCAGAGACGCTTTATAGGTGCC	RRQRRFIGA	1.91	ND	Qin et al., 2008 (4)
DQ417112	SRZ/03	AGGAGACAGAAACGCTTTATAGGCGCC	RRQKRFIGA	1.70	D	Qin et al., 2008 (4)
DQ227245	SBZ/02	GGGAGACAGGGGCGCCTTATAGGCGCC	GRQGRLIGA	1.86	D	Qin et al., 2008 (4)
DQ228923	SY/03	GGGAGACAGGGGCGCCTTATAGGCGCC	GRQGRLIGA	0.46	D	Qin et al., 2008 (4)
DQ228922	SQZ/04	AGGAGACAAAAACGCTTTATAGGCGCT	RRQKRFIGA	2.00	ND	Qin et al., 2008 (4)
DQ227250	QE01/99	GGGAGACAGGGGCGCCTTATAGGCGCC	GRQGRLIGA	1.81	D	Qin et al., 2008 (4)
DQ363532	JS05/03	GGGAGACAGGGGCGCCTTATAGGCGCC	GRQGRLIGA	1.75	D	Qin et al., 2008 (4)
AY 028995	Ch-A7/96	AAGAGACAGAAACGCTTTATAGGTGCC	KRQKRFIGA	1.89	ND	Yu et al., 2001 (5)
AF 358785	Ch/98-1	AAGAGGCAGAAACGATTTATAGGTGCC	KRQKRFIGA	1.48	ND	Yu et al., 2001 (5)
AF 364835	Ch/98-3	AGGAGACAAAAACGCTTTATAGGTGCT	RRQKRFIGA	1.89	ND	Yu et al., 2001 (5)
AF 358787	Ch/99	AGGAGACAAAAACGCTTTATAGGTGCT	RRQKRFIGA	1.84	ND	Yu et al., 2001 (5)
AF 358788	Ch/2000	AGGAGACAAAAACGCTTTATAGGTGCT	RRQKRFIGA	1.83	ND	Yu et al., 2001 (5)
AF 358786	TW/2000	AGGAGACAAAAACGCTTTATCGGTGCC	RRQKRFIGA	1.89	ND	Yu et al., 2001 (5)
AF109880	CH 62/96 (Switzerland)	AGGAGACAGAAACGCTTTATAGGTGCC	RRQKRFIGA	1.69	ND	Yu et al., 2001 (5)
AF109883	CZ 3898/96 (Czech Republic)	AGGAGACAGAAACGCTTTATAGGTGCC	RRQKRFIGA	1.69	ND	Yu et al., 2001 (5)
AF083966	TW/95-9	AGGAGACAGAAACGCTTTATAGGTGCC	RRQKRFIGA	1.68	ND	Yu et al., 2001 (5)
AF109881	DE R143/95 (Germany)	AGGAGACAGAAACGCTTTATAGGTGCC	RRQKRFIGA	1.86	ND	Yu et al., 2001 (5)
AF109885	GB 1168/84 (United Kingdo	GGGAGGCAGAAACGCTTTATAGGTGCC	GRQKRFIGA	0.85	ND	Yu et al., 2001 (5)

	m)					
AF136773	Q-GB 506/97 (United Kingdom)	AGAAGACAGAAACGCTTTATAGGTGCC	RRQKRFIGA	1.65	ND	Yu et al., 2001 (5)
M17710	Italien/45 (Italy)	AGGAGACAGAGACGCTTTATAGGTGCC	RRQRRFIGA	1.85	ND	Yu et al., 2001 (5)
M24702	Herts 33 (United Kingdom)	AGGAGACAGAGACGCTTTATAGGTGCC	RRQRRFIGA	1.99	ND	Yu et al., 2001 (5)
M23407	Texas GB/48 (United States)	AGGAGACAGAAACGCTTTATAGGCGCC	RRQKRFIGA	1.74	D	Yu et al., 2001 (5)
M24692	D26/76 (Japan)	GGGAAACAGGGACGTCTTATAGGCGCC	GKQGRLIGA	0	D	Yu et al., 2001 (5)
M21881	AUS Victoria /32	AGGAGACAGAAACGCTTTATAGGTGCT	RRQKRFIGA	1.66	ND	Yu et al., 2001 (5)
AF079172	Ch-F48E9/44	AGGAGGCAGAGACGCTTTATAGGTGCC	RRQRRFIGA	1.89	ND	Yu et al., 2001 (5)
AF083970	TW/95-3	AGGAGACAGAAACGATTTATAGGTGCT	RRQKRFIGA	1.68	ND	Yu et al., 2001 (5)
FJ772463	2415-580-Burkina Faso-2008	AGGAGACAGAAACGCTTTATAGGTGCC	RRQKRFIGA	1.69	ND	Susta et al., 2014 (6)
JN872165	Niger/06	AGACGACAGAAACGCTTTATAGGTGCA	RRQKRFIGA	1.84	ND	Susta et al., 2014 (6)
KF442614	Nigeria/06	AGGAGACAGAGACGCTTTATAGGTGCT	RRQRRFIGA	1.90	ND	Susta et al., 2014 (6)
KF442615	SA/08	AGGAGACAAAAACGCTTTATAGGTGCA	RRQKRFIGA	1.91	ND	Susta et al., 2014 (6)
AY390291	PB9601	AAGAGGCAGAAACGGTTTATAGGTGCC	KRQKRFIGA	1.44	ND	Liu et al., 2006 (7)
AY390288	YZ9712	AAGAGGCAGAAACGGTTTATAGGTGCC	KRQKRFIGA	1.53	ND	Liu et al., 2006 (7)
AY390289	NC9701	AAGAGGCAGAAACGGTTTATAGGTGCC	KRQKRFIGA	1.38	ND	Liu et al., 2006 (7)
AY390292	SZa9803	AAGAGGCAGAAACGGTTTATAGGTGCC	KRQKRFIGA	1.44	ND	Liu et al., 2006 (7)

AY390293	SZb9803	AAGAGGCAGAAACGGTTTATAGGTGCC	KRQKRFIGA	1.44	ND	Liu et al., 2006 (7)
AY390290	NP9904	AAGAGGCAGAAACGGTTTATAGGTGCC	KRQKRFIGA	1.38	ND	Liu et al., 2006 (7)
AY390314	PK9910	GGGAGACAGGGGCGCCTTATAGGCGCC	GRQGRLIGA	0.41	D	Liu et al., 2006 (7)
DQ217683	NDV03-018	AAGAGACAGAAACGCTTTATAGGTGCC	KRQKRFIGA	1.84	ND	Liu et al., 2006(7)
DQ217685	NDV03-020	AGGAGACAAAAACGCTTTATAGGTGCT	RRQKRFIGA	1.34	ND	Liu et al., 2006(7)
DQ217709	NDV03-044	AAGAGACAGAAACGCTTTATAGGTGCC	KRQKRFIGA	1.80	ND	Liu et al., 2006(7)
DQ217716	NDV03-051	AAGAGGCAGAAACGGTTTATAGGTGCC	KRQKRFIGA	1.44	ND	Liu et al., 2006 (7)
DQ439884	NDV05-027	AGGAGGCAGAAGCGCTTTATAGGTGCC	RRQKRFIGA	1.31	ND	Liu et al., 2006 (7)
DQ439885	NDV05-028	AAGAGGCAGAAACGGTTTATAGGTGCC	KRQKRFIGA	1.43	ND	Liu et al., 2006 (7)
DQ439886	NDV05-029	AAGAGGCAGAAACGGTTCATAGGTGCC	KRQKRFIGA	1.48	ND	Liu et al., 2006 (7)
GQ288381	cormorant/US(CA)/D9704285/1997	AGGAGACAGAAACGTTTTGTAGGTGCT	RRQKRFVGA	1.41	ND	Rue et al., 2010 (8)
GU332663	cormorant/US/506/2006	AGGAGACAGAAACGTTTTGTAGGTGCT	RRQKRFVGA	1.49	ND	Rue et al., 2010(8)
GU332661	cormorant/US/502/2008	AAGAGACAGAAACGTTTTGTAGGTGCT	KRQKRFVGA	1.36	ND	Rue et al., 2010(8)
GU332662	cormorant/US/503/2008	AAGAGACAGAAACGTTTTGTAGGTGCT	KRQKRFVGA	1.54	ND	Rue et al., 2010(8)

GU332657	cormorant/US/498/2008	AAGAGACAGAAACGTTTTGTAGGTGCT	KRQKRFVGA	1.39	ND	Rue et al., 2010(8)
GU332655	G/2008/US/284019481	AAGAGACAGAAACGTTTTGTAGGTGCT	KRQKRFVGA	1.45	ND	Rue et al., 2010 (8)
FJ705459	G/1998/CA/254847453	AGGAGACAGAAACGTTTTGTAGGTGCT	RRQKRFVGA	1.53	ND	Rue et al., 2010 (8)
FJ705461	G/1995/CA/254847457	AGGAGACAGAAACGTTTTGTAGGTGCT	RRQKRFVGA	1.6	ND	Rue et al., 2010 (8)
DQ833289	G/1995/CA/111052705	AGGAGACAGAAACGTTTTGTAGGTGCT	RRQKRFVGA	1.71	ND	Rue et al., 2010 (8)
DQ837552	G/1995/CA/254847455	AGGAGACAGAAACGTTTTGTAGGTGCT	RRQKRFVGA	1.59	ND	Rue et al., 2010 (8)
GQ288387	G/1992/US/253317820	AGGAGACAGAAACGTTTTGTAGGTGCT	RRQKRFVGA	1.39	ND	Rue et al., 2010(8)
AF503643	G/1992/US/20799674	AGGAGACAGAAACGTTTTGTAGGTGCT	RRQKRFVGA	1.51	ND	Rue et al., 2010(8)
FJ705463	G/2005/US/254847461	AAGAGACAGAAACGTTTTGTAGGTGCT	KRQKRFVGA	1.53	ND	Rue et al., 2010(8)
FJ705462	G/2003/US/254847459	AGGAGACAGAAACGTTTTGTAGGTGCT	RRQKRFVGA	1.41	ND	Rue et al., 2010(8)
AY142244	Northants 72	AGGAGACAGAAACGCTTTGTAGGTGCC	RRQKRFVGA	1.91	ND	Alexander et al., 1974 (9)
U22286	Texas219	AGGAGACAAAAGCGCTTTATAGGTGCC	RRQKRFIGA	1.81	ND	Alexander et al., 1974 (9)
AY024333	3015/V00	AGGAGACAGAGACGTTTTATAGGTGCC	RRQRRFIGA	1.8	ND	Cattoli et al., 2001(10)
AF506767	98/103	AGGAGGCAGAAGCGCTTTATAGGTGCC	RRQKRFIGA	0.75	ND	Meulemans et al., 2002 (11)
AF506768	98/169	AGGAGGCAGAAGCGCTTTATAGGTGCC	RRQKRFIGA	0.57	ND	Meulemans et al., 2002 (11)
FJ597613	D/ZJ/1/04	GGGAAACAGGGACGCCTTATAGGCGCT	GKQGRLIGA	0.2	D	Liu et al., 2009 (12)
FJ597550	D/AH/11/04	GAGAAACAGGGACGCCTTATAGGCGCT	EKQGRLIGA	0.2	D	Liu et al., 2009 (12)
FJ597617	D/HN/34/05	GAGAAACAGGGACGCCTTATAGGCGCT	EKQGRLIGA	0.2	D	Liu et al., 2009 (12)

FJ597553	D/HN/35/05	GAGAAACAGGGACGCCTTATAGGCGCT	EKQGRLIGA	0.2	D	Liu et al., 2009 (12)
FJ597618	D/HN/36/05	GAGAAACAGGGACGCCTTATAGGCGCT	EKQGRLIGA	0.2	D	Liu et al., 2009 (12)
AF456438	ZJ/1/00/Go	AGGAGACAAAAACGCTTTATAGGTGCT	RRQKRFIGA	1.89	ND	Wan et al., 2004(13)
AF456435	JS/1/97/Go	AGGAGGCAGAGACGCTTTATAGGTGCC	RRQRRFIGA	1.84	ND	Wan et al., 2004 (13)
AF378250	QH-1/79	AGGAGACAGAAGCGCTTTATAGGTGCC	RRQKRFIGA	1.93	ND	Wan et al., 2004 (13)
GQ168924	F48E8	AGGAGGCAGAGACGCTTTATAGGTGCC	RRQRRFIGA	1.99	ND	Wan et al., 2004 (13)
JN682190	chicken/CP/Islamabad2/2010	AGGAGACAGAAACGCTTTATAGGTGCC	RRQKRFIGA	1.6	ND	Munir et al., 2012 (14)
JN682189	chicken/CP/Rawalpindi2/2010	AGGAGACAGAAACGTTTTATAGGTGCC	RRQKRFIGA	1.67	ND	Munir et al., 2012 (14)
JN682188	chicken/CP/Attock/2010	AGAAAACAGAAACGTTTTATAGGTGCC	RKQKRFIGA	1.75	ND	Munir et al., 2012 (14)
JN682187	chicken/BYP/Rawalpindi/2010	AGGAGACAGAAACGCTTTATAGGTGCC	RRQKRFIGA	1.5	ND	Munir et al., 2012 (14)
JN682186	chicken/CP/Islamabad1/2010	AGGAGACAGAAACGCTTTATAGGTGCC	RRQKRFIGA	1.5	ND	Munir et al., 2012(14)
JN682185	chicken/CP/Rawalpindi1/2010	AGGAGACAGAAACGCTTTATAGGTGCC	RRQKRFIGA	1.72	ND	Munir et al., 2012 (14)
JN682184	chicken/BYP/Lahore/2010	AGGAGACAGAAACGCTTTATAGGTGCC	RRQKRFIGA	1.5	ND	Munir et al., 2012(14)

JN682191	chicken/CP/Islamabad3/2010	AGGAGACAGAAACGCTTTATAGGTGCC	RRQKRFIGA	1.72	ND	Munir et al., 2012 (14)
DQ227248	APMV-1/chicken/Benin/373GC/2009	AGGAGACAAAAACGCTTTATAGGTGCT	RRQKRFIGA	1.51	ND	Samuel et al., 2013 (15)
JX546244	NDV/chicken/Benin/376GT/2009	AGGAGACAGAAACGCTTTATAGGTGCC	RRQKRFIGA	1.62	ND	Samuel et al., 2013 (15)
JX546247	APMV-1/chicken/Benin/488MT/2009	AGGAGACAGAAACGCTTTATAGGTGCC	RRQKRFIGA	1.62	ND	Samuel et al., 2013 (15)
JX546245	NDV/chicken/Benin/463MT/2009	AGACGACGGAAACGTTTTGTAGGTGCA	RRRKRFVGA	1.65	ND	Samuel et al., 2013 (15)
JX390609	NDV/chicken/Togo/AKO18/2009	AGGAGACGAAAACGCTTTATAGGTGCC	RRRKRFIGA	1.65	ND	Samuel et al., 2013 (15)
EF418787	TW-06/06	AGGAGACAAAAACGCTTTATAGGTGCC	RRQKRFIGA	1.84	ND	Lien et al., 2007 (16)
EF418789	TW-03/05	AGGAGACAAAAACGCTTTATAGGTGCC	RRQKRFIGA	1.80	ND	Lien et al., 2007 (16)
DQ054375	TW-204/03	AGGAGACAAAAACGCTTTATAGGTGCC	RRQKRFIGA	1.81	ND	Lien et al., 2007 (16)

DQ054372	TW-005/03	AGGAGACAAAAACGCTTTATAGGTGCC	RRQKRFIGA	1.76	ND	Lien et al., 2007 (16)
EF418785	TW-10/06	AGGAGAAAAAAACGCTTTATAGGTGCC	RRKKRFIGA	1.83	ND	Lien et al., 2007(16)
EF418786	TW-09/06	AGGAGAAAAAAACGCTTTATAGGTGCC	TW-09/06	1.78	ND	Lien et al., 2007(16)
EF418788	TW-04/06	AGGAGAAAAAAACGCTTTATAGGTGCC	RRKKRFIGA	1.71	ND	Lien et al., 2007(16)
EF418790	TW-02/05	AGGAGAAAAAAACGCTTTATAGGTGCC	RRKKRFIGA	1.75	ND	Lien et al., 2007(16)
EF418791	TW-01/05	AGGAGAAAAAAACGCTTTATAGGTGCC	RRKKRFIGA	1.85	ND	Lien et al., 2007(16)
DQ074638	TW-817/04	AGGAGAAAAAAACGCTTTATAGGTGCC	RRKKRFIGA	1.88	ND	Lien et al., 2007(16)
DQ074636	TW-799/04	AGGAGAAAAAAACGCTTTATAGGTGCC	RRKKRFIGA	1.88	ND	Lien et al., 2007(16)
DQ074641	TW-692/04	AGGAGAAAAAAACGCTTTATAGGTGCC	RRKKRFIGA	1.89	ND	Lien et al., 2007(16)
AF217084		GGGAAACAGGGACGTCTTATAGGCGCC	GKQGRLIGA	0.39	D	Ke et al. 2010(17)
D00243		GGGAAACAGGGACGCCTTATAGGCGCC	GKQGRLIGA	0.39	D	Ke et al. 2010(17)
M24692		GGGAAACAGGGACGTCTTATAGGCGCC	GKQGRLIGA	0.00	D	Ke et al. 2010 (17)
X04719		AGGAGACAGAAACGCTTTATAGGCGCC	RRQKRFIGA	1.75	D	Ke et al. 2010(17)
M23407		AGGAGACAGAAACGCTTTATAGGCGCC	RRQKRFIGA	1.74	D	Ke et al. 2010(17)
AF077761	LaSota	GGGAGACAGGGGCGCCTTATAGGCGCC	GRQGRLIGA	0.31	D	Ke et al. 2010(17)
U22266		GGGAGACAGGGGCGCCTTATAGGCGCC	GRQGRLIGA	0.13	D	Ke et al. 2010(17)
M21881		AGGAGACAGAAACGCTTTATAGGTGCT	RRQKRFIGA	1.66	ND	Ke et al. 2010(17)
AF083970	TW/95-3	AGGAGACAGAAACGATTTATAGGTGCT	RRQKRFIGA	1.68	ND	Ke et al. 2010(17)

M24702		AGGAGACAGAGACGCTTTATAGGTGCC	RRQRRFIGA	1.99	ND	Ke et al. 2010(17)
U22274	CA1083	AGGAGACAGAAACGCTTTATAGGTGCT	RRQKRFIGA	1.8	ND	Ke et al. 2010(17)
AF458013	JS-2/98	AGGAGACAGAAACGCTTCATAGGTGCC	RRQKRFIGA	1.88	ND	Ke et al. 2010(17)
AY028995	CH-A7/96	AAGAGACAGAAACGCTTTATAGGTGCC	KRQKRFIGA	1.89	ND	Ke et al. 2010(17)
AF458010	JS-3/00	AAGAGACAGAAACGCTTTATAGGTGCC	KRQKRFIGA	1.74	ND	Ke et al. 2010(17)
DQ898538	TW-02-301	AGGAGACAAAAACGCTTTATAGGTGCC	RRQKRFIGA	1.75	ND	Ke et al. 2010(17)
AF456442	JS/5/01/Go	AGGAGACAAAAACGCTTTATAGGTGCT	RRQKRFIGA	1.81	ND	Ke et al. 2010(17)
AF364835	Ch/98-3	AGGAGACAAAAACGCTTTATAGGTGCT	RRQKRFIGA	1.89	ND	Ke et al. 2010(17)
AF358786	TW/2000	AGGAGACAAAAACGCTTTATCGGTGCC	RRQKRFIGA	1.89	ND	Ke et al. 2010(17)
AF234031	TW/99-156	AGGAGACAAAAACGCTTTATAGGTGCC	RRQKRFIGA	1.79	ND	Ke et al. 2010(17)
AF234032	TW/99-157	AGGAGACAAAAACGCTTTATAGGTGCC	RRQKRFIGA	1.79	ND	Ke et al. 2010(17)
AF326523	TW/99-165	AGGAGACAAAAACGCTTTATAGGTGCC	RRQKRFIGA	1.79	ND	Ke et al. 2010(17)
AF326525	TW/99-173	AGGAGACAAAAACGCTTTATAGGTGCC	RRQKRFIGA	1.73	ND	Ke et al. 2010(17)
AF326524	TW/99-174	AGGAGACAAAAACGCTTTATAGGTGCC	RRQKRFIGA	1.73	ND	Ke et al. 2010(17)
DQ898539	TW-02-300B	AGGAGACAAAAACGCTTTATAGGTGCC	RRQKRFIGA	1.73	ND	Ke et al. 2010(17)
DQ898538	TW-02-301	AGGAGACAAAAACGCTTTATAGGTGCC	RRQKRFIGA	1.75	ND	Ke et al. 2010(17)
DQ898537	TW-02-302	AGGAGACAAAAACGCTTTATAGGTGCC	RRQKRFIGA	1.75	ND	Ke et al. 2010(17)
DQ898536	TW-02-303	AGGAGACAAAAACGCTTTATAGGTGCC	RRQKRFIGA	1.76	ND	Ke et al. 2010(17)

DQ898535	TW-02-312	AGGAGACAAAAACGCTTTATAGGTGCC	RRQKRFIGA	1.54	ND	Ke et al. 2010(17)
DQ898534	TW-03-328	AGGAGACAAAAACGCTTTATAGGTGCC	RRQKRFIGA	1.75	ND	Ke et al. 2010(17)
DQ898533	TW-03-329	AGGAGACAAAAACGCTTTATAGGTGCC	RRQKRFIGA	1.75	ND	Ke et al. 2010(17)
DQ898532	TW-03-330	AGGAGACAAAAACGCTTTATAGGTGCC	RRQKRFIGA	1.75	ND	Ke et al. 2010(17)
DQ898530	TW-03-332	AGGAGAAAAAAACGCTTTATAGGTGCC	RRKKRFIGA	1.74	ND	Ke et al. 2010(17)
DQ898529	TW-03-333	AGGAGAAAAAAACGCTTTATAGGTGCC	RRKKRFIGA	1.75	ND	Ke et al. 2010(17)
DQ898528	TW-04-CB8	AGGAGAAAAAAACGCTTTATAGGTGCC	RRKKRFIGA	1.74	ND	Ke et al. 2010(17)
DQ898527	TW-04-338	AGGAGACAAAAACGCTTTATAGGTGCC	RRQKRFIGA	1.74	ND	Ke et al. 2010(17)
DQ898526	TW-04-340	AGGAGACAAAAACGCTTTATAGGTGCC	RRQKRFIGA	1.75	ND	Ke et al. 2010(17)
DQ898525	TW-05-346	AGGAGACAAAAACGCTTTATAGGTGCC	RRQKRFIGA	1.75	ND	Ke et al. 2010(17)
DQ898524	TW-05-347	AGGAGACAAAAACGCTTTATAGGTGCC	RRQKRFIGA	1.73	ND	Ke et al. 2010(17)
DQ898523	TW-05-348	AGGAGACAAAAACGCTTTATAGGTGCC	RRQKRFIGA	1.74	ND	Ke et al. 2010(17)
DQ898522	TW-06-126A3	AGGAGAAAAAAACGCTTTATAGGTGCC	RRKKRFIGA	1.75	ND	Ke et al. 2010(17)
DQ898521	TW-06-223	AGGAGAAAAAAACGCTTTATAGGTGCC	RRKKRFIGA	1.71	ND	Ke et al. 2010(17)
AY508514	F48E9	AGGAGGCAGAGACGCTTTATAGGTGCC	RRQRRFIGA	1.99	ND	Ke et al. 2010(17)
AF458009	FJ-1/85	AGGAGGCAGAGACGCTTTATAGGTGCC	RRQRRFIGA	1.89	ND	Ke et al. 2010(17)
AF458022	JS-1/97	AGGAGGCAGAGACGCTTTATAGGTGCC	RRQRRFIGA	1.90	ND	Ke et al. 2010(17)

AB853926	JP/Osaka/2440/69	AGGAGACAGAAACGCTTTATAGGTGCC	RRQKRFIGA	1.8	ND	Umali et al. 2013 (18)
AB853927	JP/Ibaraki/SG106/99	AGGAGACAAAAACGCTTTATAGGTGCC	RRQKRFIGA	1.7	ND	Umali et al. 2013(18)
AB853928	JP/Ibaraki/SM87/87	AGGAGACAGAAACGCTTTATAGGTGCC	RRQKRFIGA	1.7	ND	Umali et al., 2013 (18)
AB853929	JP/Miyagi/AGT/02	AGGAGACAAAAACGCTTTATAGGTGCC	RRQKRFIGA	1.8	ND	Umali et al., 2013(18)
AB853930	JP/Chiba/BY7/02	AGGAGACAAAAACGCTTTATAGGTGCC	RRQKRFIGA	1.7	ND	Umali et al., 2013(18)
AB853931	JP/Chiba/BY103/01	AGGAGACAAAAACGCTTTATAGGTGCC	RRQKRFIGA	1.8	ND	Umali et al., 2013(18)
AB853932	JP/Ibaraki/IS2/02	AGGAGACAAAAACGCTTTATAGGTGCC	RRQKRFIGA	1.9	ND	Umali et al., 2013(18)
AB853933	JP/Ibaraki/IS5/02	AGGAGACAAAAACGCTTTATAGGTGCC	RRQKRFIGA	1.8	ND	Umali et al , 2013(18)
AF438364	NZ51/76	GGGAAACAGGGACGTCTTATAGGAGCC	GGKQGRLIGA	0.11	D	Stanislawek et al., 2002 (19)
AF438365	NZ131/76	GGGAAACAGGGACGTCTTATAGGAGCC	GKQGRLIGA	0.02	D	Stanislawek et al., 2002(19)
NZ10/97	NZ983/01	GGGAAACAGGGACGTCTTATAGGAGCC	GKQGRLIGA	0.16	D	Stanislawek et al., 2002(19)
AF438379	NZ9/97	GGGAAACAGGGACGTCTTATAGGAGCC	GKQGRLIGA	0.10	D	Stanislawek et al., 2002(19)
AF438378	NZ8/97	GGGAAACAGGGACGTCTTATAGGAGCC	GKQGRLIGA	0.10	D	Stanislawek et al., 2002(19)
AF438377	NZ7/97	GGGAAACAGGGACGTCTTATAGGAGCC	GKQGRLIGA	0.00	D	Stanislawek et al., 2002(19)
AF438376	NZ6/97	GGGAAACAGGGACGTCTTATAGGAGCC	GKQGRLIGA	0.00	D	Stanislawek et al., 2002(19)
AF438375	NZ5/97	GGGAAACAGGGACGTCTTATAGGAGCC	GKQGRLIGA	0.00	D	Stanislawek et al., 2002(19)
AF438374	NZ4/97	GGGAAACAGGGACGTCTTATAGGAGCC	GKQGRLIGA	0.00	D	Stanislawek et al., 2002(19)
AF438373	NZ3/97	GGGAAACAGGGACGTCTTATAGGAGCC	GKQGRLIGA	0.00	D	Stanislawek et al., 2002(19)

AF438372	NZ2/97	GGGAAACAGGGACGTCTTATAGGAGCC	GKQGRLIGA	0.00	D	Stanislawek et al., 2002(19)
AF438371	NZ8043/95	GGGAAACAGGGACGTCTTATAGGAGCC	GKQGRLIGA	0.00	D	Stanislawek et al., 2002(19)
AF438370	NZ1/97	GAACGGCAGGGGCGTTTGGTGGGGGCA	ERQGRLVGA	0.00	ND	Stanislawek et al., 2002(19)
AF438369	NZ3528/78	GGGAAACAGGGACGTCTTATAGGAGCC	GKQGRLIGA	0.04	D	Stanislawek et al., 2002(19)
AF438367	NZ7579/78	GGGAAACAGGGACGTCTTATAGGAGCC	GKQGRLIGA	0.00	D	Stanislawek et al., 2002(19)
AF438366	NZ132/76	GGGAAACAGGGACGTCTTATAGGAGCC	GKQGRLIGA	0.14	D	Stanislawek et al., 2002(19)
AY935491	98-1154	AGGAGACAGGGGCGTCTTATAGGCGCC	RRQGRLIGA	0.47	D	Kattenbelt et al., 2006(20)
AY935495	99-0868hi	AGGAGACAGGGGCGTCTTATAGGCGCC	RRQGRLIGA	0.51	D	Kattenbelt et al., 2006(20)
AY935496	99-0868lo	AGGAGACAGAGGCGTCTTATAGGCGCC	RRQRRLIGA	1.38	D	Kattenbelt et al., 2006(20)
AY935497	99-1997PR-32	AGGAGACAGGGGCGTTTTATAGGCGCC	RRQGRFIGA	1.68	D	Kattenbelt et al., 2006(20)
AY935492	98-1249	AGGAGACAGAGGCGTTTTATAGGCGCC	RRQRRFIGA	1.68	D	Kattenbelt et al., 2006(20)
AY935493	98-1252	AGGAGACAGAGGCGTTTTATAGGCGCC	RRQRRFIGA	1.78	D	Kattenbelt et al., 2006(20)
AY935494	99-0655	AGGAGACAGAGGCGTTTTATAGGCGCC	RRQRRFIGA	1.43	D	Kattenbelt et al., 2006(20)
AY935498	99-1435	AGGAGACAGAGGCGTTTTATAGGCGCC	RRQRRFIGA	1.69	D	Kattenbelt et al., 2006(20)
AY935489	01-1108	AGGAGACAGGGGCGTCTTATAGGCGCC	RRQGRLIGA	0.6	D	Kattenbelt et al., 2006(20)
AY935490	02-1334	AGGAGACAGAGGCGTTTTATAGGCGCC	RRQRRFIGA	1.61	D	Kattenbelt et al., 2006(20)

Supplementary Table 9: Comparison of Nucleotide pattern, Amino acid motif, MDT and *BSAH*1 digestion

GenBank no.	Name Of Isolate	Nucleotide pattern (334-360)	Amino acid (112-120)	MDT	*BSAH*1 digestion	Reference
FJ597614	D_ZJ_2_04	GGGAAACAGGGACGCCTTATAGGCGCT	GKQGRLIGA	96	D	Liu et al., 2009(12)
FJ597615	D_ZJ_3_04	GAGAAACAGGGACGCCTTATAGGCGCT	EKQGRLIGA	96	D	Liu et al., 2009(12)
FJ597568	D_ZJ_4_04	GGGAAACAGGGACGCCTTATAGGCGCT	GKQGRLIGA	96	D	Liu et al., 2009(12)
FJ597603	D_AH_5_04	GAGAAACAGGGACGCCTTATAGGCGCT	EKQGRLIGA	96	D	Liu et al., 2009(12)
FJ597604	D_AH_6_04	GAGAAACAGGGACGCCTTATAGGCGCT	EKQGRLIGA	96	D	Liu et al., 2009(12)
FJ597548	D_AH_7_04	GGAGAGAAACAGGGACGCCTTATAGGCGCT	EKQGRLIGA	96	D	Liu et al., 2009(12)
FJ597569	D_ZJ_8_04	GGGAAACAGGGACGCCTTATAGGCGCT	GKQGRLIGA	96	D	Liu et al., 2009(12)
FJ597570	D_ZJ_9_04	GGGAAACAGGGACGCCTTATAGGCGCT	GKQGRLIGA	96	D	Liu et al., 2009(12)
FJ597549	D_AH_10_04	GAGAAACAGGGACGCCTTATAGGCGCT	EKQGRLIGA	96	D	Liu et al., 2009(12)
FJ597571	D_ZJ_12_04	GGGAAACAGGGACGCCTTATAGGCGCT	GKQGRLIGA	96	D	Liu et al., 2009(12)
FJ597605	D_AH_13_04	GAGAAACAGGGACGCCTTATAGGCGCT	EKQGRLIGA	96	D	Liu et al., 2009(12)
FJ597551	D_AH_14_04	GAGAAACAGGGACGCCTTATAGGCGCT	EKQGRLIGA	96	D	Liu et al., 2009(12)
FJ597552	D_AH_15_04	GAGAAACAGGGACGCCTTATAGGCGCT	EKQGRLIGA	96	D	Liu et al., 2009(12)
FJ597554	D_JS_7_05	GAGAAACAGGGACGCCTTATAGGCGCT	EKQGRLIGA	96	D	Liu et al., 2009(12)
FJ597555	D_JS_8_05	GAGAAACAGGGACGCCTTATAGGCGCT	EKQGRLIGA	96	D	Liu et al., 2009(12)
FJ597556	D_JS_9_05	GAGAAACAGGGACGCCTTATAGGCGCT	EKQGRLIGA	96	D	Liu et al., 2009(12)

FJ597557	D_JS_10_05	GAGAAACAGGGACGCCTTATAGGCGCT	EKQGRLIGA	96	D	Liu et al., 2009(12)
FJ597566	D_SD_11_05	GAGAAACAGGGACGCCTTATAGGCGCT	EKQGRLIGA	96	D	Liu et al., 2009(12)
FJ597558	D_JS_12_05	GGGAAACAGGGACGCCTTATAGGCGCT	GKQGRLIGA	96	D	Liu et al., 2009(12)
FJ597567	D_SD_13_05	GAGAAACAGGGACGCCTTATAGGCGCT	EKQGRLIGA	96	D	Liu et al., 2009(12)
FJ597611	D_JS_14_05	GAGAAACAGGGACGCCTTATAGGCGCT	EKQGRLIGA	96	D	Liu et al., 2009(12)
FJ597612	D_SD_15_05	GGGAAACAGGGACGCCTTATAGGCGCT	GKQGRLIGA	96	D	Liu et al., 2009(12)
FJ597619	D_HN_37_05	GAGAAACAGGGACGCCTTATAGGCGCT	EKQGRLIGA	96	D	Liu et al., 2009(12)
FJ597620	D_JS_39_05	GGGAAACAGGGACGCCTTATAGGCGCT	GKQGRLIGA	96	D	Liu et al., 2009(12)
FJ597606	D_JS_40_05	GGGAAACAGGGACGCCTTATAGGCGCT	GKQGRLIGA	96	D	Liu et al., 2009(12)
FJ597607	D_JS_41_05	GGGAAACAGGGACGCCTTATAGGCGCT	GKQGRLIGA	96	D	Liu et al., 2009(12)
FJ597608	D_JS_42_05	GGGAAACAGGGACGCCTTATAGGCGCT	GKQGRLIGA	96	D	Liu et al., 2009(12)
FJ597559	D_JS_43_05	GGGAAACAGGGACGCCTTATAGGCGCT	GKQGRLIGA	96	D	Liu et al., 2009(12)
FJ597560	D_JS_44_05	GGGAAACAGGGACGCCTTATAGGCGCT	GKQGRLIGA	96	D	Liu et al., 2009(12)
FJ597561	D_JS_45_05	GGGAAACAGGGACGCCTTATAGGCGCT	GKQGRLIGA	96	D	Liu et al., 2009(12)
FJ597562	D_JS_46_05	GGGAAACAGGGACGCCTTATAGGCGCT	GKQGRLIGA	96	D	Liu et al., 2009(12)
FJ597563	D_JS_47_05	GGGAAACAGGGACGCCTTATAGGCGCT	GKQGRLIGA	96	D	Liu et al., 2009(12)
FJ597564	D_JS_48_05	GGGAAACAGGGACGCCTTATAGGCGCT	GKQGRLIGA	96	D	Liu et al., 2009(12)
FJ597565	D_JS_49_05	GGGAAACAGGGACGCCTTATAGGCGCT	GKQGRLIGA	96	D	Liu et al., 2009(12)
FJ597616	D_ZJ_50_05	GAGAAACAGGGACGCCTTATAGGCGCT	EKQGRLIGA	96	D	Liu et al., 2009(12)

FJ597609	D_JS_51_05	GGGAAACAGGGACGCGTTATAGGCGCT	GKQGRVIGA	96	ND	Liu et al., 2009(12)
FJ597572	D_ZJ_52_05	GAGAAACAGGGACGCCTTATAGGCGCT	EKQGRLIGA	96	D	Liu et al., 2009(12)
FJ597610	D_JS_53_05	GGGAAACAGGGACGCCTTATAGGCGCT	GKQGRLIGA	96	D	Liu et al., 2009(12)
JX546275	NDV/chicken/Togo/AKO11/2009	AGGAGACAAAAACGCTTTATAGGTGCC	RRQKRFIGA	48	ND	Samuel et al., 2013 (15)
JX546274	NDV/duck/Togo/AKC14/2009	AGGAGACAAAAACGCTTTATAGGTGCC	RRQKRFIGA	48	ND	Samuel et al., 2013 (15)
JX546273	NDV/chicken/Togo/AKC12/2009	AGGAGACAAAAACGCTTTATAGGTGCC	RRQKRFIGA	48	ND	Samuel et al., 2013 (15)
JX546272	NDV/duck/Togo/GBO5/2009	AGGAGACAAAAACGCTTTATAGGTGCC	RRQKRFIGA	48	ND	Samuel et al., 2013 (15)
JX546271	NDV/chicken/Benin/846GC/2009	AGGAGACAGAAACGCTTTATAGGTGCC	RRQKRFIGA	48	ND	Samuel et al., 2013 (15)
JX546270	NDV/chicken/Benin/378GT/2009	AGGAGACAGAAACGCTTTATAGGTGCC	RRQKRFIGA	56	ND	Samuel et al., 2013 (15)
JX546269	NDV/chicken/Benin/379GT/2009	AGGAGACAGAAACGCTTTATAGGTGCC	RRQKRFIGA	48	ND	Samuel et al., 2013 (15)
JX546268	NDV/chicken/Benin/770GT/2009	AGGAGACGAAAACGCTTTATAGGTGCC	RRRKRFIGA	48	ND	Samuel et al., 2013 (15)
JX546267	NDV/chicken/Benin/349GC/2009	AGGAGACAGAAACGCTTTATAGGTGCC	RRQKRFIGA	48	ND	Samuel et al., 2013 (15)
JX546265	NDV/chicken/Benin/372GC/2009	AGGAGACAGAAACGCTTTATAGGTGCC	RRQKRFIGA	56	ND	Samuel et al., 2013 (15)

JX546264	NDV/chicken/Benin/380GC/2009	AGGAGACAGAAACGCTTTATAGGTGCC	RRQKRFIGA	48	ND	Samuel et al., 2013 (15)
JX546263	NDV/chicken/Benin/847GC/2009	AGGAGACAGAAACGCTTTATAGGTGCC	RRQKRFIGA	48	ND	Samuel et al., 2013 (15)
JX546262	NDV/chicken/Benin/382GT/2009	AGGAGACAGAAACGCTTTATAGGTGCC	RRQKRFIGA	64	ND	Samuel et al., 2013 (15)
JX546261	NDV/chicken/Benin/479MT/2009	AGGAGACAGAAACGCTTTATAGGTGCC	RRQKRFIGA	64	ND	Samuel et al., 2013 (15)
JX546260	NDV/chicken/Benin/442MT/2009	AGGAGGCAGAAACGCTTCATAGGTGCC	RRQKRFIGA	48	ND	Samuel et al., 2013 (15)
JX546246	NDV/chicken/Benin/474MC/2009	AGGAGGCAGAAACGCTTCATAGGTGCC	RRQKRFIGA	48	ND	Samuel et al., 2013 (15)
JX546259	NDV/chicken/Benin/415MC/2009	AGGAGGCAGAAACGCTTCATAGGTGCC	RRQKRFIGA	48	ND	Samuel et al., 2013 (15)
JX546258	NDV/chicken/Benin/480MT/2009	AGGAGACAGAAACGCTTTATAGGTGCC	RRQKRFIGA	48	ND	Samuel et al., 2013 (15)
JX546257	NDV/chicken/Benin/476MT/2009	AGGAGACAGAAACGCTTTATAGGTGCC	RRQKRFIGA	56	ND	Samuel et al., 2013 (15)
JX546256	NDV/chicken/Benin/467MC/2009	AGGAGGCAGAAACGCTTCATAGGTGCC	RRQKRFIGA	48	ND	Samuel et al., 2013 (15)
JX546255	NDV/chicken/Benin/432MT/2009	AGGAGGCAGAAACGCTTCATAGGTGCC	RRQKRFIGA	48	ND	Samuel et al., 2013 (15)
AF234030	TW/99-154	AGGAGACAGAAACGCTTTATAGGTGCC	RRQKRFIGA	48	ND	Ke et al. 2010 (17)

DQ363535	SF02	AGGAGACAAAAACGCTTTATAGGTGCT	RRQKRFIGA	48	ND	Ke et al. 2010 (17)
AB512601	TW/07-401	AGGAGACAAAAACGCTTTATAGGTGCC	RRQKRFIGA	44	ND	Ke et al. 2010 (17)
AB512602	TW/07-402	AGGAGACAAAAACGCTTTATAGGTGCC	RRQKRFIGA	44	ND	Ke et al. 2010 (17)
AB512603	TW/07-403	AGGAGACAAAAACGCTTTATAGGTGCC	RRQKRFIGA	44	ND	Ke et al. 2010 (17)
AB512604	TW/07-405	AGGAGACAAAGACGCTTTATAGGTGCC	RRQRRFIGA	44	ND	Ke et al. 2010 (17)
AB512605	TW/08-406	AGGAGACAAAAACGCTTTATAGGTGCC	RRQKRFIGA	44	ND	Ke et al. 2010 (17)
AB512606	TW/08-407	AGGAGACAAAAACGCTTTATAGGTGCC	RRQKRFIGA	44	ND	Ke et al. 2010 (17)
AB512607	TW/08-408	AGGAGACAAAAACGCTTTATAGGTGCC	RRQKRFIGA	44	ND	Ke et al. 2010 (17)
AB512608	TW/08-412	AGGAGACAAAAACGCTTTATAGGTGCC	RRQKRFIGA	44	ND	Ke et al. 2010 (17)
AB512614	TW/08-02	AGGAGACAAAAACGCTTTATAGGTGCC	RRQKRFIGA	44	ND	Ke et al. 2010 (17)
AB512615	TW/08-05	AGGAGACAAAAACGCTTTATAGGTGCC	RRQKRFIGA	44	ND	Ke et al. 2010 (17)
AB512616	TW/08-07	AGGAGACAAAAACGCTTTATAGGTGCC	RRQKRFIGA	44	ND	Ke et al. 2010 (17)
JQ007344	KNU-C/HL/09	GGGAAACAGGGACGTCTTATAGGCGCC	GKQGRLIGA	96	D	Choi et al. 2012 (21)
JQ007350	KNU-D/GP09/09	GGGAAACAGGGACGTCTTATAGGCGCC	GKQGRLIGA	96	D	Choi et al. 2012 (21)
JQ007348	KNU-C/PC/09	GGGAAACAGGGACGTCTTATAGGCGCC	GKQGRLIGA	96	D	Choi et al. 2012 (21)
JQ007346	KNU-C/JG/09	GGGAAACAGGGACGCCTTATAGGCGCC	GKQGRLIGA	96	D	Choi et al. 2012 (21)
JQ007349	KNU-C/SN/09	GGGAAACAGGGACGTCTTATAGGCGCC	GKQGRLIGA	96	D	Choi et al. 2012 (21)

JQ007345	KNU-C/JA/09	GGGAGACAGGGGCGCCTTATAGGCGCC	GRQGRLIGA	96	D	Choi et al. 2012 (21)
JQ007343	KNU-C/HC/09	GGGAAACAGGGACGTCTTATAGGCGCC	GKQGRLIGA	96	D	Choi et al. 2012 (21)
JQ007351	KNU-D/HC/09	GGGAAACAGGGACGTCTTATAGGCGCC	GKQGRLIGA	96	D	Choi et al. 2012 (21)
JQ007352	KNU-D/WSL/09	GGGAGACAGGGGCGCCTTATAGGCGCC	GRQGRLIGA	96	D	Choi et al. 2012 (21)
JQ007347	KNU-C/JS/09	GGGAAACAGGGACGTCTTATAGGCGCC	GKQGRLIGA	96	D	Choi et al. 2012 (21)

Supplementary Table 10: Comparison of Nucleotide pattern, Amino acid motif, vaccine strain NDV and *BSAH*1 digestion

**GenBank no.**	**Name Of Isolate**	**Nucleotide pattern (334-360)**	**Amino acid (112-120)**	***BSAH*1 digestion**
EF534703	Ulster	GGGAAACAGGGACGCCTTATAGGCGCC	GKQGRLIGA	D
JN872152	Ulster	GGGAAACAGGGACGCCTTATAGGCGCC	GKQGRLIGA	D
U22290	Ulster	GGGAAACAGGGACGCCTTATAGGCGCC	GKQGRLIGA	D
D00243	Ulster	GGGAAACAGGGACGCCTTATAGGCGCC	GKQGRLIGA	D
AY562991	chicken/N. Ireland/Ulster/67	GGGAAACAGGGACGCCTTATAGGCGCC	GKQGRLIGA	D
JN634863	Queensland V4	GGGAAACAGGGACGTCTTATAGGCGCC	GKQGRLIGA	D
AF217084	V4 Queensland	GGGAAACAGGGACGTCTTATAGGCGCC	GKQGRLIGA	D
JX524203	NDV V4	GGGAAACAGGGACGTCTTATAGGCGCC	GKQGRLIGA	D
AY225110	HB92 isolate V4	GGGAGACAGGGGCGCCTTATAGGCGCC	GRQGRLIGA	D
NC_002617	Hitchner B1	GGGAGACAGGGGCGCCTTATAGGCGCC	GRQGRLIGA	D
AF375823	B1 isolate Takaaki	GGGAGACAGGGGCGCCTTATAGGCGCC	GRQGRLIGA	D
AF309418	B1	GGGAGACAGGGGCGCCTTATAGGCGCC	GRQGRLIGA	D
JN872150	B1	GGGAGACAGGGGCGCCTTATAGGCGCC	GRQGRLIGA	D

U22266	B1	GGGAGACAGGGGCGCCTTATAGGCGCC	GRQGRLIGA	D
AF542767	97-0788 smuggled B1 vaccine strain	GGGAGACAGGGGCGCCTTATAGGCGCC	GRQGRLIGA	D
AF542786	B1-wey	GGGAGACAGGGGCGCCTTATAGGCGCC	GRQGRLIGA	D
EF440343	F strain	GGGAGACAGGGGCGCCTTATAGGCGCC	GRQGRLIGA	D
EF440344	LaSota	GGGAGACAGGGGCGCCTTATAGGCGCC	GRQGRLIGA	D
DQ195265	Lasota	GGGAGACAGGGGCGCCTTATAGGTGCC	GRQGRLIGA	D
JN863120	LaSota	GGGAGACAGGGGCGCCTTATAGGCGCC	GRQGRLIGA	D
JF950510	LaSota	GGGAGACAGGGGCGCCTTATAGGTGCC	GRQGRLIGA	D
AF077761	LaSota	GGGAGACAGGGGCGCCTTATAGGCGCC	GRQGRLIGA	D
AY845400	LaSota	GGGAGACAGGGGCGCCTTATAGGCGCC	GRQGRLIGA	D
AJ249525	LaSota	GGGAGACAGGGGCGCCTTATAGGCGCC	GRQGRLIGA	D
U22292	LaSota	GGGAGACAGGGGCGCCTTATAGGCGCC	GRQGRLIGA	D
Y16170	H strain	AGGAGACAGAGACGCTTTATAGGTGCC	K R Q K R F I G A	ND
AF224505	Mukteswar	AGGAGACAGAGACGCTTTATAGGTGCC	R R Q R R F I G A	ND
AY117021	Mukteswar/IN	AGGAGACAGAGACGCTTTATAGGTGCC	R R Q R R F I G A	ND
AF224505	S (Primary Industries Enterprise Ltd) Mukteswar (SINGAPORE)	AGGAGACAGAGACGCTTTATAGGTGCC	R R Q R R F I G A	ND
AY117022	MUKTESWAK *SUBOTICA a.d*(FR Yugoslavia)	AGGAGACAGAGACGCTTTATAGGTGCC	R R Q R R F I G A	ND
AY117023	TB-MUKTESWAR (Zemun-FR Yugoslavia)	AGGAGACAGAGACGCTTTATAGGTGCC	R R Q R R F I G A	ND
AF224504	H(Ph/80) Herefordshir	AGGAGACAGAGACGCTTTATAGGTGCC	R R Q R R F I G A	ND

	e, Phylaxia,Budapest, Hungary			
AY170136	H(Ph/02) Herefordshire,CEVA-Phylaxia,Budapest,Hungary	AGGAGACAGAGACGCTTTATAGGTGCC	R R Q R R F I G A	ND
AF224503	H/W(Herefordshire)Weybridge	AGGAGACAGAGACGCTTTATAGGTGCC	R R Q R R F I G A	ND
AJ249527	R2B	AGGAGACAGAAACGCTTTATAGGCGCC	R R Q K R F I G A	D
JX316216	R2B	AGGAGACAGAAACGCTTTATAGGCGCC	R R Q K R F I G A	D
AJ249527	R2B	AGGAGACAGAAACGCTTTATAGGCGCC	R R Q K R F I G A	D
JN872154	Beaudette C	AGGAGACAGAAACGCTTTATAGGCGCC	R R Q K R F I G A	D
X04719	Beaudette C	AGGAGACAGAAACGCTTTATAGGCGCC	RRQKRFIGA	D
JN863121	Roakin	AGGAGACAGAAACGCTTTATAGGCGCC	R R Q K R F I G A	D
U22284	Roakin	AGGAGACAGAAACGCTTTATAGGCGCC	RRQKRFIGA	D
AY289000	chicken/USA/Roakin/48	AGGAGACAGAAACGCTTTATAGGCGCC	RRQKRFIGA	D
EF440345	Komarov	AGGAGACAGAAACGCTTTATAGGCGCC	R R Q K R F I G A	D
AY170137	Komarov/45/LK	AGGAGACAGAAACGCTTTATAGGCGCC	RRQKRFIGA	D

D- Digested, ND- Not digested

Reference

1. Weingartl, H.M., Riva, J. and Kumthekar, P. (2003) Molecular characterization of avian paramyxovirus 1 isolates collected from cormorants in Canada from 1995 to 2000. *Journal of clinical microbiology*, **41**, 1280-1284.
2. Vijayarani, K., Muthusamy, S., Tirumurugaan, K.G., Sakthivelan, S.M. and Kumanan, K. (2010) Pathotyping of a Newcastle disease virus isolated from peacock (Pavo cristatus). *Tropical animal health and production*, **42**, 415-419.

3. Terregino, C., Cattoli, G., Grossele, B., Bertoli, E., Tisato, E. and Capua, I. (2003) Characterization of Newcastle disease virus isolates obtained from Eurasian collared doves (Streptopelia decaocto) in Italy. *Avian pathology : journal of the W.V.P.A*, **32**, 63-68.
4. Qin, Z.M., Tan, L.T., Xu, H.Y., Ma, B.C., Wang, Y.L., Yuan, X.Y. and Liu, W.J. (2008) Pathotypical characterization and molecular epidemiology of Newcastle disease virus isolates from different hosts in China from 1996 to 2005. *Journal of clinical microbiology*, **46**, 601-611.
5. Yu, L., Wang, Z., Jiang, Y., Chang, L. and Kwang, J. (2001) Characterization of newly emerging Newcastle disease virus isolates from the People's Republic of China and Taiwan. *Journal of clinical microbiology*, **39**, 3512-3519.
6. Susta, L., Hamal, K.R., Miller, P.J., Cardenas-Garcia, S., Brown, C.C., Pedersen, J.C., Gongora, V. and Afonso, C.L. (2014) Separate evolution of virulent newcastle disease viruses from Mexico and Central America. *Journal of clinical microbiology*, **52**, 1382-1390.
7. Liu, H., Wang, Z., Son, C., Wang, Y., Yu, B., Zheng, D., Sun, C. and Wu, Y. (2006) Characterization of pigeon-origin Newcastle disease virus isolated in China. *Avian diseases*, **50**, 636-640.
8. Rue, C.A., Susta, L., Brown, C.C., Pasick, J.M., Swafford, S.R., Wolf, P.C., Killian, M.L., Pedersen, J.C., Miller, P.J. and Afonso, C.L. (2010) Evolutionary changes affecting rapid identification of 2008 Newcastle disease viruses isolated from double-crested cormorants. *Journal of clinical microbiology*, **48**, 2440-2448.
9. Alexander, D.J. and Allan, W.H. (1974) Newcastle disease virus pathotypes. *Avian pathology : journal of the W.V.P.A*, **3**, 269-278.
10. Cattoli, G., Manvell, R.J., Tisato, E., Banks, J. and Capua, I. (2001) Characterization of Newcastle disease viruses isolated in Italy in 2000. *Avian pathology : journal of the W.V.P.A*, **30**, 465-469.
11. Meulemans, G., van den Berg, T.P., Decaesstecker, M. and Boschmans, M. (2002) Evolution of pigeon Newcastle disease virus strains. *Avian pathology : journal of the W.V.P.A*, **31**, 515-519.
12. Liu, X., Wang, X., Wu, S., Hu, S., Peng, Y., Xue, F. and Liu, X. (2009) Surveillance for avirulent Newcastle disease viruses in domestic ducks (Anas platyrhynchos and Cairina moschata) at live bird markets in Eastern China and characterization of the viruses isolated. *Avian pathology : journal of the W.V.P.A*, **38**, 377-391.
13. Wan, H., Chen, L., Wu, L. and Liu, X. (2004) Newcastle disease in geese: natural occurrence and experimental infection. *Avian pathology : journal of the W.V.P.A*, **33**, 216-221.
14. Munir, M., Cortey, M., Abbas, M., Qureshi, Z.U., Afzal, F., Shabbir, M.Z., Khan, M.T., Ahmed, S., Ahmad, S., Baule, C. *et al.* (2012) Biological characterization and phylogenetic analysis of a novel genetic group of Newcastle disease virus isolated from outbreaks in commercial poultry and from backyard poultry flocks in Pakistan. *Infection, genetics and evolution : journal of molecular epidemiology and evolutionary genetics in infectious diseases*, **12**, 1010-1019.
15. Samuel, A., Nayak, B., Paldurai, A., Xiao, S., Aplogan, G.L., Awoume, K.A., Webby, R.J., Ducatez, M.F., Collins, P.L. and Samal, S.K. (2013) Phylogenetic and pathotypic characterization of newcastle disease viruses circulating in west Africa and efficacy of a current vaccine. *Journal of clinical microbiology*, **51**, 771-781.
16. Lien, Y.Y., Lee, J.W., Su, H.Y., Tsai, H.J., Tsai, M.C., Hsieh, C.Y. and Tsai, S.S. (2007) Phylogenetic characterization of Newcastle disease viruses isolated in Taiwan during 2003-2006. *Veterinary microbiology*, **123**, 194-202.
17. Ke, G.M., Yu, S.W., Ho, C.H., Chu, P.Y., Ke, L.Y., Lin, K.H., Tsai, Y.C., Liu, H.J. and Lin, M.Y. (2010) Characterization of newly emerging Newcastle disease viruses isolated during 2002-2008 in Taiwan. *Virus research*, **147**, 247-257.

18. Umali, D.V., Ito, H., Suzuki, T., Shirota, K., Katoh, H. and Ito, T. (2013) Molecular epidemiology of Newcastle disease virus isolates from vaccinated commercial poultry farms in non-epidemic areas of Japan. *Virology journal*, **10**, 330.
19. Stanislawek, W.L., Wilks, C.R., Meers, J., Horner, G.W., Alexander, D.J., Manvell, R.J., Kattenbelt, J.A. and Gould, A.R. (2002) Avian paramyxoviruses and influenza viruses isolated from mallard ducks (Anas platyrhynchos) in New Zealand. *Archives of virology*, **147**, 1287-1302.
20. Kattenbelt, J.A., Stevens, M.P. and Gould, A.R. (2006) Sequence variation in the Newcastle disease virus genome. *Virus research*, **116**, 168-184.
21. Choi, K.S., Lee, E.K., Jeon, W.J., Kwon, J.H., Lee, J.H. and Sung, H.W. (2012) Molecular epidemiologic investigation of lentogenic Newcastle disease virus from domestic birds at live bird markets in Korea. *Avian diseases*, **56**, 218-223.
